# Fast response of complementary electrochromic device based on WO_3_/NiO electrodes

**DOI:** 10.1038/s41598-020-65191-x

**Published:** 2020-05-21

**Authors:** Po-Wen Chen, Chen-Te Chang, Tien-Fu Ko, Sheng-Chuan Hsu, Ke-Ding Li, Jin-Yu Wu

**Affiliations:** 1 Division of Physics, Institute of Nuclear Energy Research, Taoyuan County, 32546 Taiwan; 2 Department of Materials Science and Engineering, National Cheng Kung University, Tainan, 70101 Taiwan

**Keywords:** Chemistry, Chemistry, Environmental sciences, Environmental sciences

## Abstract

Nanoporous structures have proven as an effective way for enhanced electrochromic performance by providing a large surface area can get fast ion/electron transfer path, leading to larger optical modulation and fast response time. Herein, for the first time, application of vacuum cathodic arc plasma (CAP) deposition technology to the synthesis of WO_3_/NiO electrode films on ITO glass for use in fabricating complementary electrochromic devices (ECDs) with a ITO/WO_3_/LiClO_4_-Perchlorate solution/NiO/ITO structure. Our objective was to optimize electrochromic performance through the creation of electrodes with a nanoporous structure. We also examined the influence of WO_3_ film thickness on the electrochemical and optical characteristics in terms of surface charge capacity and diffusion coefficients. The resulting 200-nm-thick WO_3_ films achieved ion diffusion coefficients of (7.35 × 10^−10^ (oxidation) and 4.92 × 10^−10^ cm^2^/s (reduction)). The complementary charge capacity ratio of WO_3_ (200 nm thickness)/NiO (60 nm thickness) has impressive reversibility of 98%. A demonstration ECD device (3 × 4 cm^2^) achieved optical modulation (ΔT) of 46% and switching times of 3.1 sec (coloration) and 4.6 sec (bleaching) at a wavelength of 633 nm. In terms of durability, the proposed ECD achieved ΔT of 43% after 2500 cycles; i.e., 93% of the initial device.

## Introduction

Over the past decades, electrochromic devices (ECDs) have been used in energy efficient buildings, optical information displays, variable-reflectance mirrors, switchable mirrors, and electronic papers^1–5^. Electrochromism materials change their optical characteristics (transmittance, reflectance, and absorption) reversibly through applying a dc voltage^6^. Electrochromism has attracted much attention because it could provide a tremendous promising application in energy-saving smart windows. Smart windows based on electrochromic (EC) materials easily control the indoor sunlight and solar heat and can be effectively reduced the heating or cooling loads of building interiors^6^. A wide variety of electrochromic materials have been developed, including metal oxides^7–9^, small organic molecules^10^, and conductive polymer thin films^11–13^. Complementary ECDs are composed of anodic and cathodic coloring materials in a five-layer structure. A pair of transparent conducting layers sandwich an ionic conduction layer (electrolyte) in contact with an electrochromic (EC) layer and an ion storage (complementary) layer^14–16^. Tungsten oxide (WO_3_) is known as one of the most popular cathodic coloration material and nickel oxide (NiO) as typical anodic coloration material, which has been intensively investigated^14–17^.

WO_3_ film is a transition metal oxide, which can be reversibly switched between colorless and blue under positive or negative voltage, respectively^18,19^. The chemical reaction underlying the electrochromic behavior of WO_3_ films is based on reversible oxidation/reduction reactions induced by the electrochemical double injection/extraction of positive ions (lithium or proton) and electrons into/out of the host WO_3_ lattice in the transition from W^5+^ to W^5+^ ^19,20^. WO_3_ films have been fabricated in a variety of nanostructures, including nano-rods^19,21,22^, nanosheets^20^, and nanotrees^23^. Note however that a dense structure, low diffusion coefficient, and/or long diffusion length for ion insertion tend to hinder optical modulation performance. By increasing the contact area between the electrode and electrolyte, nanoporous WO_3_ structures reduce the diffusion path of ions and providing channels to facilitate the rapid transfer of ion/electrons, resulting in particularly good electrochromic and electrochemical performance^19,20,23^. Electrochromic WO_3_ films have been fabricated using a variety of methods, including sputtering^24–27^, chemical vapor deposition (CVD)^28–30^, spray pyrolysis^31,32^, thermal evaporation^33^, electron-beam deposition^34^, and sol-gel^35–37^ methods. Lee *et al*.^19^ recently reported on the synthesis of uniform WO_3_ nano-rods, resulting in films with fast coloration/bleaching times (28.8/4.5 sec at 633 nm). Zhang *et al*.^23^ used thermal annealing in the synthesis of WO_3_ electrodes within a one-dimensional structure, which resulted in good CE (43.6 cm^2^/C), and fast coloration/bleaching times (7.6/4.2 sec at 633 nm).

Different above technology, in the current study, we used cathodic arc plasma (CAP) technology to fabricate WO_3_/NiO films with a porous surface structure with the aim of enhancing electrochromic performance and accelerating switching speeds^38–41^. We also examined the degree to which the thickness of WO_3_ layers affects the electrochemical and optical properties of ECDs.

## Results

### Electrochromic mechanism for NiO-WO_3_ system

**Figure 1:**
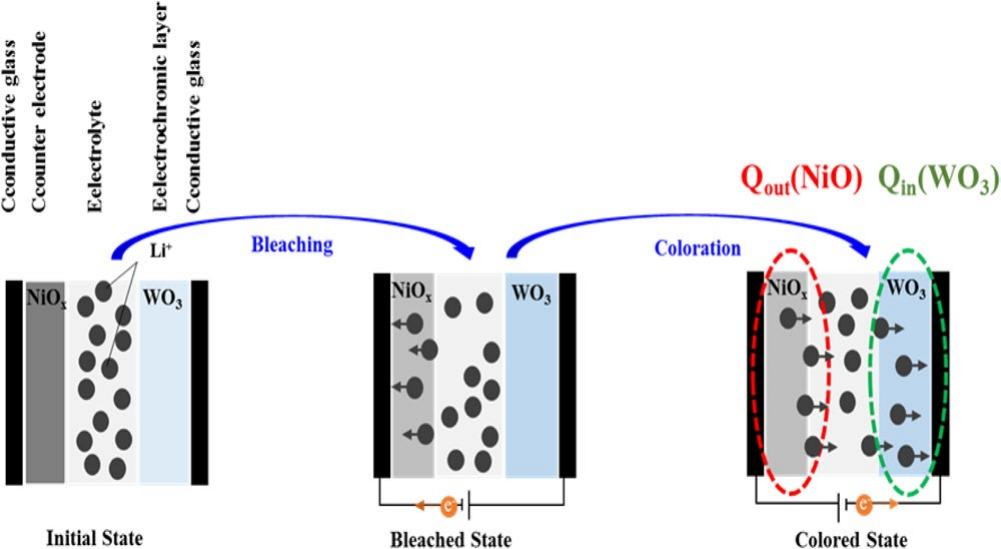
Working principles for ECD. Schematic diagram of coloration and bleaching process of with WO_3_ electrochromic layer and NiO counter layer.

As shown in Fig. 1(a–c), the application of voltage (an electric field) to the device causes positive ions to move toward the electric field, while the electrons move in the opposite direction. The movement of ions (electrons) into the electrochromic (ion storage) layers is responsible for the coloration (bleaching) of the ECDs. The underlying physics involved in the electrochromic reactions can be represented using the following the redox equations:

1 $${{\rm{WO}}}_{3}({\rm{bleaching}})+x({M}^{+}+{{\rm{e}}}^{-})\overleftarrow{\to }{M}_{x}{{\rm{WO}}}_{3}({\rm{coloration}}),$$

where *M* indicates the lithium ions (Li^+^) or hydrogen ions (H^+^) ions. The WO_3_ thin film changes from transparent to deep blue under the effects of electron insertion (i.e., photo-effected intervalence electron transfer from W^6+^ to W^5+^ sites). The electrochromic mechanism governing the behavior of Li^+^ ions against ion insertion/extraction in the NiO electrode can be represented using the following the redox equations:

2 $${{\rm{N}}{\rm{i}}{\rm{O}}}_{x}+y({M}^{+}+{{\rm{e}}}^{-})\to {M}_{y}{{\rm{N}}{\rm{i}}{\rm{O}}}_{x},{M}_{y}{{\rm{N}}{\rm{i}}{\rm{O}}}_{x}({\rm{c}}{\rm{o}}{\rm{l}}{\rm{o}}{\rm{r}}{\rm{a}}{\rm{t}}{\rm{i}}{\rm{o}}{\rm{n}}){M}_{y-z}{{\rm{N}}{\rm{i}}{\rm{O}}}_{x}({\rm{b}}{\rm{l}}{\rm{e}}{\rm{a}}{\rm{c}}{\rm{h}}{\rm{i}}{\rm{n}}{\rm{g}})+z({M}^{+}+{{\rm{e}}}^{-}).$$

The reduction of Ni^3+^ to Ni^2+^ leads to the bleaching of the NiO film (during the cathodic scan), and coloration of NiO film via the oxidation of Ni^2+^ to Ni^3+^ (in the reverse process). Continuously applying negative voltage to the NiO electrode (ion storage layer) causes the insertion of electrons and Li^+^ ions leading to the oxidation of Ni^2+^ to Ni^3+^, with the result that the coloration state is dominant. In Fig. 1(c), for test step, to understand surface charge capacity for WO_3_/NiO electrode films, which were integrated CA curves defined as both intercalation surface charges (Q_*in*_) and extraction surface charges (Q_*out*_). Here, the complementary surface charge capacity ratio *R* is defined as the insertion WO_3_ electrode divided by the extraction NiO electrode.

### WO_3_/ITO and NiO/ITO films: surface charge capacity ratio

The complementary ECD in the current study included two electrochromic electrodes, as in thin-film batteries. Thus, we calculated the surface charge capacity ratio of the electrodes in both directions. We first sought to elucidate the electrochemical and energy storage properties of the WO_3_/ITO/glass by constructing three electrode cells, which comprised a working electrode (WO_3_ film on ITO/glass), a counter-electrode (Pt mesh) and a reference electrode (Ag/AgCl) in 0.5 M LiClO_4_/Perchlorate (LiClO_4_/PC) solution.

**Figure 2:**
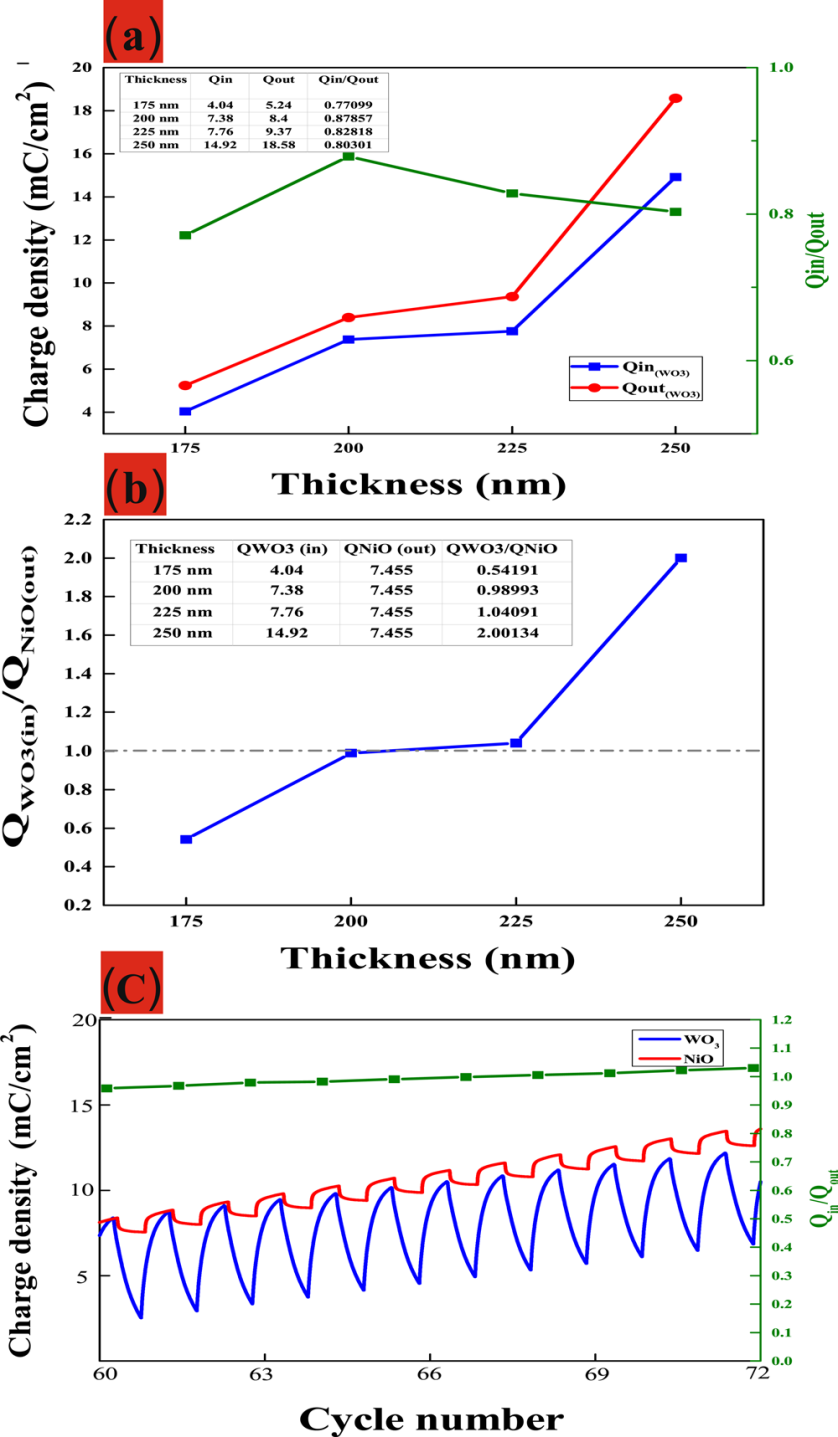
(Color online) (**a**) Surface charge capacity of WO_3_/ITO/glass for Q_*in*_ and Q_*out*_ (**b**) Complementary charge capacity ration *R* = Q_*in*_ (WO_3_)/Q_*out*_(NiO) (**c**) Reversibility of *R*.

The surface charge capacity of the WO_3_ layers was based on integral to CA curves was carried with from −1.5 to 0.3 *V* versus AgCl/Ag in intercalation surface charges (Q_*in*_) and extraction surface charges (Q_*out*_). As shown in Fig. 2(a), the 200-nm-thick WO_3_ electrode returned the following values: Q_*in*_ (7.38 mC cm^−2^), Q_*out*_ (8.4 mC cm^−2^), and reversibility, Q_*in*_/Q_*out*_ is about 87% better than the other samples. Here, complementary charge capacity ratio *R* is defined as the intercalation WO_3_ electrode divided by the extraction NiO electrode according to following equation:

3 $$R={Q}_{in}({{\rm{WO}}}_{3})/{Q}_{out}({\rm{NiO}}),$$

where Q_*in*_ (WO_3_) is the surface charge capacity of the WO_3_ electrode during intercalation and Q_*out*_ (NiO) is the surface charge capacity of the NiO electrode during the extraction process. Q_*out*_ (NiO) was carried with from 0.7 *V* versus Ag/AgCl and observed Q_*out*_ (NiO) value is 7.45 mC cm^−2^. As shown in Fig. 2(b), we also assessed the degree to which the thickness of the WO_3_ layer (175 nm, 200 nm, 225 nm and 250 nm) affected the charge capacity ratio when using an NiO/ITO/glass electrode of 60 nm in thickness. With the charge capacity of the NiO electrode fixed at 7.45 mC cm^−2^, the *R* values varied as a function of WO_3_ thickness, as follows: 175 nm (0.54), 200 nm (0.98), 225 nm (1.04), and 250 nm (2.0). Note that under these conditions, the sample with a 200 nm-thick layer of WO_3_ achieved reversibility of nearly 98%. Figure 2(c) shows the reversibility of *R* = WO_3_ (200 nm)/NiO (60 nm) about 0.98~1.02 value between 60 and 72 cycles. It appears that the high R value reduced the likelihood of ion blocking, making it an ideal structure for the movement of ions at electrode/electrolyte interfaces in an electrochromic film.

### Diffusion coefficient and transmittance optical modulation as a function of WO_3_/ITO film thickness

**Figure 3:**
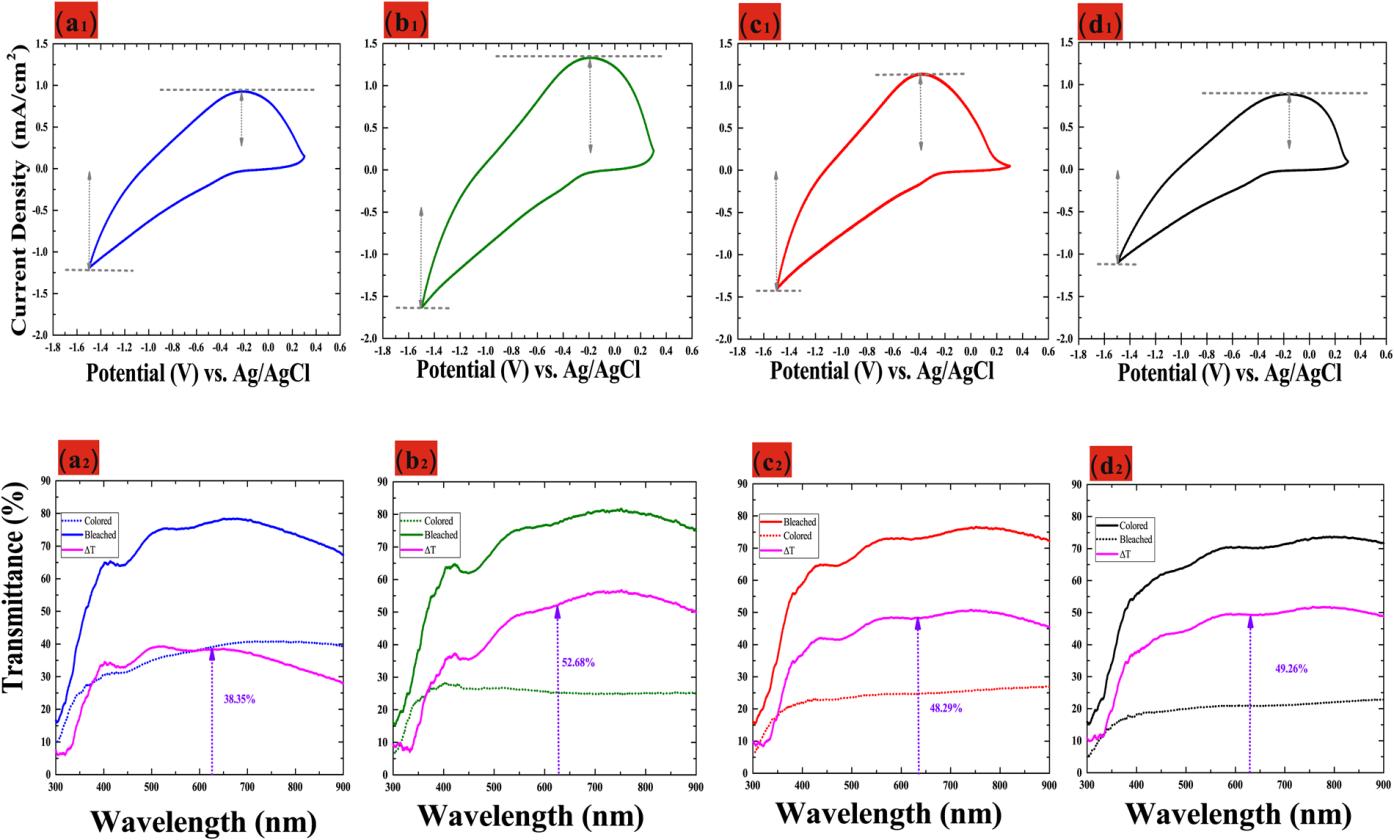
**(a**_1_**,b**_1_,c_1_,**d**_1_ _**1,**__**4**_**)** CV curves of WO_3_ films of various thicknesses during the 25th-cycle; **(a**_2_**,b**_2_,**s**_2_,**d**_2_**)** Transmittance of WO_3_ film in bleaching (solid line) and coloration (dotted line) states with various thicknesses.

Figure 3(a_1_,b_1_,c_1_,d_1_) showed cycle voltammetry (CV) curve of WO_3_/ITO films at four different thicknesses and applied the potential voltage from −1.5 V (coloration) to 0.3 V (bleaching) at a scan rate of 200 mV/s. In Fig. 3, the CV curve of WO_3_/ITO films were measured at 25^*th*^-cycle for four different thicknesses 175 nm, 200 nm, 225 nm, and 250 nm respectively. Furthermore, the diffusion coefficient *D* of Li^+^ ions during injection/extraction of ions into/out of the WO_3_ film can be calculated using the Randles–Servick Eq. (4)^19^.

4 $${J}_{p}=2.69\times {10}^{5}{n}^{\mathrm{3/2}}{C}_{0}{D}^{\mathrm{1/2}}{\nu }^{\mathrm{1/2}},$$

where *C*_0_ indicates the concentration of electrolyte solution (mol• cm^−3^); *v* is the scan rate (V• s^−1^); *D* is the diffusion coefficient (cm^2^s^−1^) and *J*_*p*_ is the peak current. Table 1 lists the values for *J*_*pc*_ (cathodic peak current density), *J*_*pa*_ (anodic peak current density), and diffusion coefficient *D* (working area 3 × 4 cm^2^). Sample 2 (with 200 nm-thick) of WO_3_/ITO films in Table 1 presented the highest ion diffusion coefficients of 7.35 × 10^−10^ cm^2^/s (oxidation) and 4.92 × 10^−10^ cm^2^/s (reduction). From Table 1, the higher diffusion coefficient indicates a larger contact area and greater porosity resulting in faster ion insertion/extraction.

**Table 1:** Diffusion coefficients of WO_3_/ITO glass of various thicknesses.

Sample	Thick(nm)	Cathodic Current(J_pc_)	Anodic Current(J_pa_)	*D* from *J*_*pc*_\J_*pa*_	
1	175	1.19 × 10^−3^	9.28 × 10^−4^	3.92 × 10^−10^	2.38 × 10^−10^
2	200	1.63 × 10^−3^	1.33 × 10^−3^	7.35 × 10^−10^	4.92 × 10^−10^
3	225	1.41 × 10^−3^	1.14 × 10^−3^	5.46 × 10^−10^	3.62 × 10^−10^
4	250	1.10 × 10^−3^	8.90 × 10^−4^	3.36 × 10^−10^	2.19 × 10^−10^

Figure 3(a_2_,b_2_,c_2_,d_2_) presents the optical transmittance spectra of WO_3_/ITO/glass between bleached and colored states at different various thicknesses (175 nm, 200 nm, 225 nm, and 250 nm). At a fixed wavelength of 633 nm, optical transmittance varied as a function of thicknesses from 38.35% to 52.68%. Note that the modulation of optical transmittance of 200-nm-thick WO_3_ film (ΔT = 52.68%) was higher than that of the other samples, as indicated by the larger enveloped area in the CV curve. Actually, the area of the CV curve is deeply related to the charge stored (capacity) at porous WO_3_ film^20^ indicates that more charges are taking part in redox reactions.

### Microstructural characteristics

**Figure 4:**
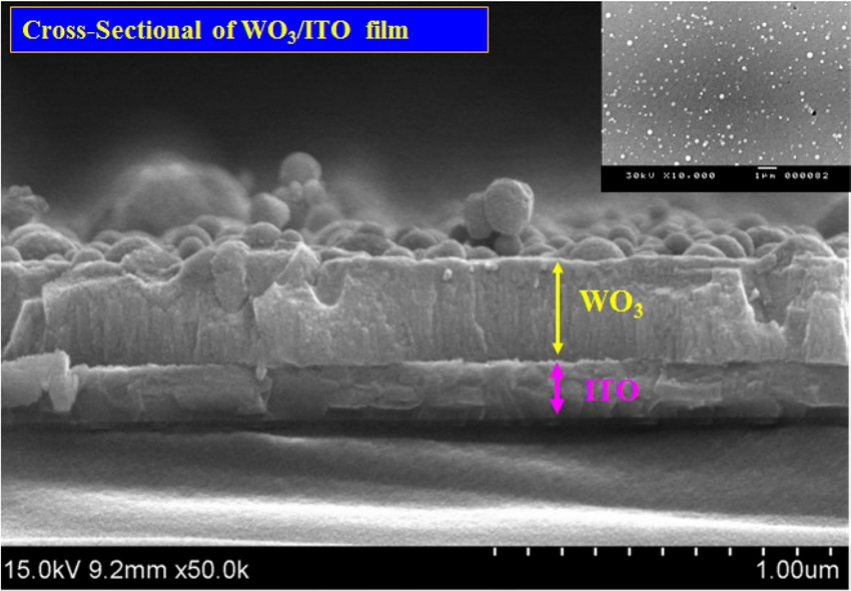
Cross-sectional morphologies of WO_3_/ITO film.

**Figure 5:**
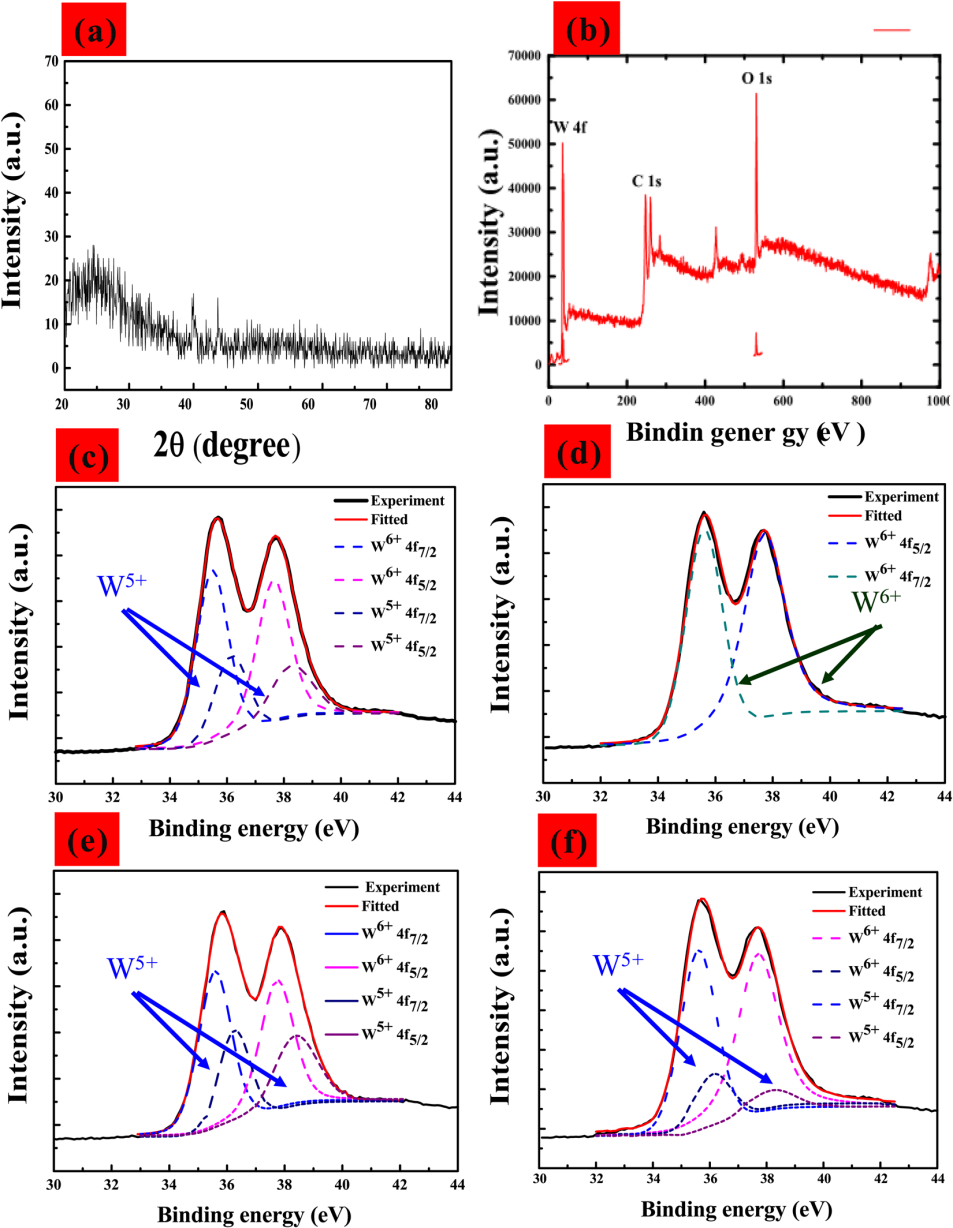
(Color online) (**a**) XRD pattern of WO_3/_glass film (**b**) Wide scanning XPS spectra of WO_3_ film (**c**,**e**) High-resolution XPS W4f spectra of coloration WO_3_ film at 200-nm-thick and 250-nm-thick. (**d**,**f**) High-resolution XPS W4f spectra of bleaching WO_3_ film at 200-nm-thick and 250-nm-thick.

The cross-sectional SEM image in Fig. 4 shows the hemispherical surface structures of the WO_3_ film deposited using CAP. Top-view SEM image of WO_3_ pattern inset of Fig. 4. Figure 5(a) presents the X-ray diffraction (XRD) patterns of the WO_3_ film deposited on a glass substrate. The porous WO_3_ film presented only one broad peak at ~23°, indicating an amorphous structure, as described in previous studies^36^. X-ray photoelectron spectroscopy (XPS) was used to analyze the chemical composition of the WO_3_ film surface. The electrochemical testing of Li_*x*_WO_3_(WO_3_:Li) samples in 0.5 M liquid-electrolyte of LiClO_4_/PC solution was performed using a three-electrode cell, comprising a working electrode (WO_3_ electrode film on ITO/glass), a counter-electrode (Pt mesh) and a reference electrode (Ag/AgCl). The color of the WO_3_/ITO/glass changed from deep blue (colored state; −1.35 *V*) to transparent (bleached state; 0.2 *V*), is accordance with the intercalation/extraction of ions (Li^+^) into/out of the WO_3_ electrode. The thickness of the WO_3_ film was shown to have considerable influence on the electrochromic properties by XPS analsis. Figure 5(c,e) show XPS spectra of W 4 f in tungsten oxide films (200 nm) and (250 nm) in coloration states. In Fig. 5(c,e), the peaks W 4f_7/2_ and W 4f_5/2_ of W^6+^ and W^5+^ that are located at binding energies of 35.17 and 37.24 eV corresponding to W4 f_5/2_ and W4 f_7/2_ of W^5+^ in the Li_*x*_WO_3_. The coloration process indicates the movement of Li^+^ ions and electrons into the WO_3_ films, such that the W^6+^ obtained an *e*^−^ to become W^5+^, resulting in a corresponding shift in the peak to a lower energy level. The content of W^5+^ in Li_*x*_WO_3_ film (i.e., *η*(W^5+^)) can be calculated using the following equation:

5 $$\eta ({{\rm{W}}}^{5+})={{\rm{W}}}^{5+}/({{\rm{W}}}^{5+}+{{\rm{W}}}^{6+})\times \mathrm{100 \% }.$$

The fitted spectrum can be separated into two gaussian doublets shown Fig. 5(c–f). In this redox reaction Eq.(5), we evaluated ions transformed from the W^6+^ to the W^5+^ state. In Fig. 5(c–e), we calculated that approximately 30% (200 nm) and 35% (250 nm) of the ions transformed from the W^6+^ to the W^5+^ state, see Table 2. This is an indication that a larger number of W^6+^ (bleaching) Li^+^ ions took part in the reduction reaction to become W^5+^ (blue). As shown in Fig. 3(b_2_,d_2_), we found that as the thickness of the film increased, Q_*in*_ (WO_3_) curve gradually increased as did the power of lithium-ion injection, optical transmittance of 200-nm-thick WO_3_ film (T_*coloration*_ = 25.12%) and 250-nm-thick WO_3_ film (T_*coloration*_ = 18.13%) at a fixed wavelength of 633 nm. Figure 5(d–f) presents high-resolution XPS spectra of W 4 f in tungsten oxide films (200 nm) and (250 nm) in bleaching states. In the W 4 f spectrum in the beached state (Fig. 5(d)), the peaks at binding energies of 35.6 and 37.7 eV correspond to W4 f_5/2_ and W4 f_7/2_ of W^6+^. Thus, we can deduce that only W^6+^ ions were present in the WO_3_ thin films in the bleached state. But when the film thickness reaches 250 nm, the film can’t completely fade, which is still light blue, which means that W^5+^ (blue) can’t completely convert to W^6+^ (bleaching).

**Table 2:** W 4 *f* peak fitting binding energy of WO_3_ film with thick of 200 and 250 nm. *FWHM-Full width half maximum.

$\frac{Thick}{(nm)}$	states	W oxidation	W 4 f	$\frac{{\rm{Bindingenergy}}}{{\boldsymbol{(}}{\bf{e}}{\bf{V}}{\boldsymbol{)}}}$	$\frac{{\rm{FWHM}}}{{\boldsymbol{(}}{\bf{e}}{\bf{V}}{\boldsymbol{)}}}$	$\frac{{\rm{Aera}}}{{\boldsymbol{(}}{\boldsymbol{ \% }}{\boldsymbol{)}}}$
200	Bleaching	W^6+^	$\frac{W4{f}_{\mathrm{7/2}}}{W4{f}_{\mathrm{5/2}}}$	$\frac{35.6}{37.7}$	$\frac{0.8}{0.8}$	100%
200	Coloration	W^6+^	$\frac{W4{f}_{\mathrm{7/2}}}{W4{f}_{\mathrm{5/2}}}$	$\frac{35.5}{37.7}$	$\frac{1.6}{1.8}$	70%
200	Coloration	W^5+^	$\frac{W4{f}_{\mathrm{7/2}}}{W4{f}_{\mathrm{5/2}}}$	$\frac{36.2}{38.4}$	$\frac{2.2}{2.2}$	30%
250	Bleaching	W^6+^	$\frac{W4{f}_{\mathrm{7/2}}}{W4{f}_{\mathrm{5/2}}}$	$\frac{35.6}{37.7}$	$\frac{1.7}{2}$	80%
250	Bleaching	W^5+^	$\frac{W4{f}_{\mathrm{7/2}}}{W4{f}_{\mathrm{5/2}}}$	$\frac{36.2}{38.2}$	$\frac{1.8}{2}$	20%
250	Coloration	W^6+^	$\frac{W4{f}_{\mathrm{7/2}}}{W4{f}_{\mathrm{5/2}}}$	$\frac{35.6}{37.8}$	$\frac{1.7}{2}$	65%
250	Coloration	W^5+^	$\frac{W4{f}_{\mathrm{7/2}}}{W4{f}_{\mathrm{5/2}}}$	$\frac{36.3}{38.4}$	$\frac{1.8}{1.9}$	35%

This is confirmed by the detection of W^5+^ in a later XPS analysis of the 250 nm bleached film. This shows that there are residual W^5+^ions in the process of fading oxidation when lithium ion is injected into the coloring reduction reaction Therefore, appropriate film thickness, will help improve the electrochromic optical modulation performance. The 200-nm-thick film contained only W^6+^ ions; however, some of the W^5+^ ions in the 250-nm-thick film did not convert into W^6+^ ions, thereby decreasing the penetration of the bleaching state, as shown in Fig. 3(b_2_,d_2_), we found optical transmittance of 200-nm-thick WO_3_ film (T_*bleaching*_ = 78.85%) and 250-nm-thick WO_3_ film (T_*bleaching*_ = 70.05%) at a fixed wavelength of 633 nm. In Fig. 5(f), we calculated that approximately 80% (250 nm) of the ions transformed from the W^5+^ to the W^6+^ state, see Table 2. Ning *et al*.^42^ claimed that lattice strain can affect the diffusion and migration of lithium ions, such that the coefficient of lithium ion diffusion decreases under the effects of pressure-induced strain. Thus, the failure of the 250-nm-thick film to recolor may be due to the thickness of the film, which extended the lithium ion migration path, such that the remaining stress hindered the transfer of lithium ions and the conversion of W^5+^ to W^6+^. Therefore, tungsten oxide film as a cathodic electrochromic layer, the film thickness should be selected appropriately, can improve modulation optical transmittance to optimization conditions.

### Coloration efficiency of WO_3_/ITO films as a function of thickness

**Figure 6:**
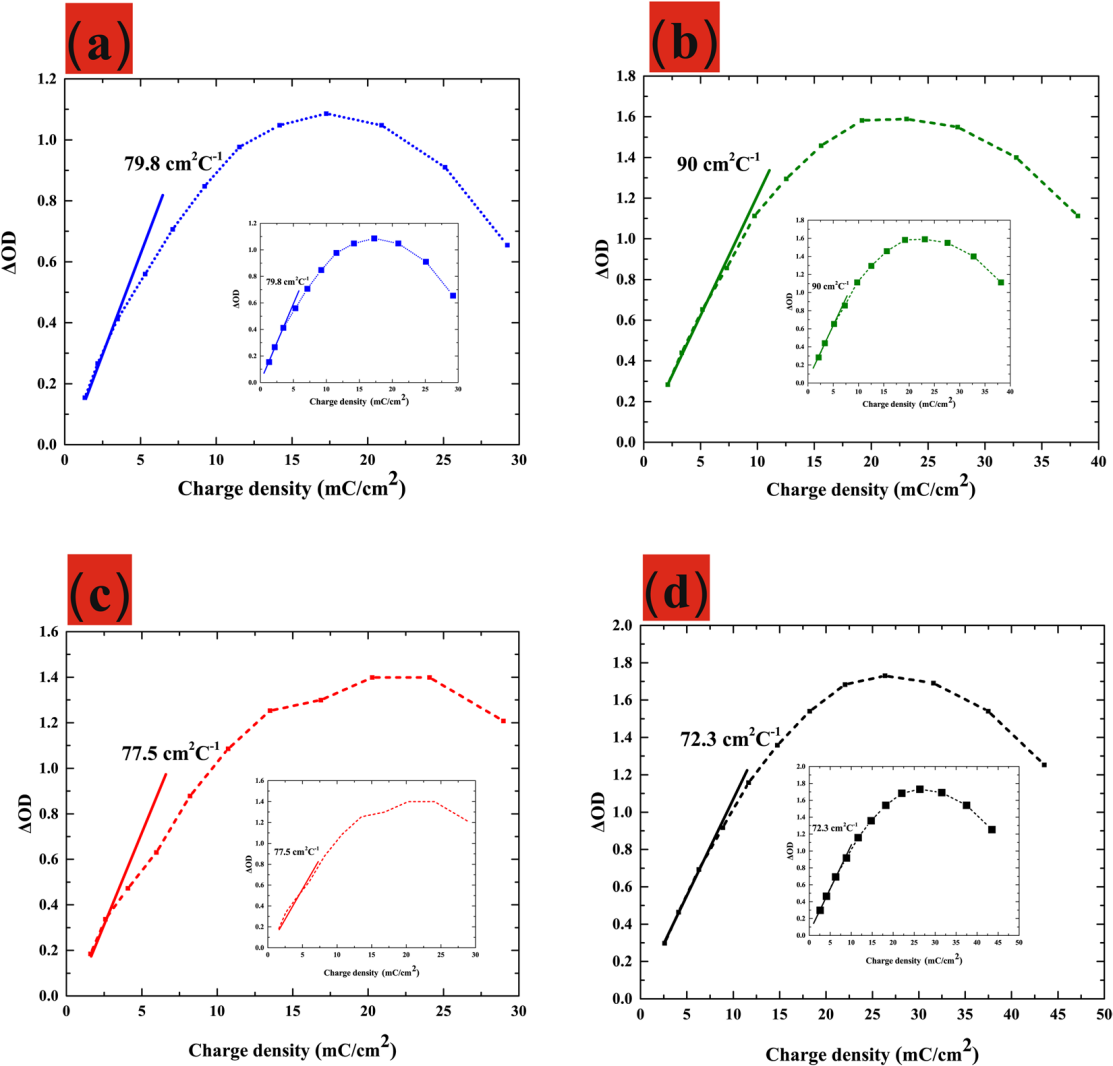
(Color online) Optical density change as a function of inserted charge for WO_3_/ITO film with various thicknesses.

Coloration efficiency (CE) is an important criterion in the evaluation of electrochromic materials. CE is defined as the optical density charge (ΔOD) per unit of inserted charge Q_*in*_ (Q_*in*_ = Q/*A*, where *A* is the ^20^:

6 $$\begin{array}{c}CE=\Delta OD/{Q}_{in}\\ \Delta OD={\rm{l}}{\rm{n}}({T}_{{\rm{b}}{\rm{l}}{\rm{e}}{\rm{a}}{\rm{c}}{\rm{h}}{\rm{i}}{\rm{n}}{\rm{g}}}/{T}_{{\rm{c}}{\rm{o}}{\rm{l}}{\rm{o}}{\rm{r}}{\rm{a}}{\rm{t}}{\rm{i}}{\rm{o}}{\rm{n}}}).\end{array}$$

where *T*_bleaching_ and *T*_coloration_ refer to the transmittance of bleaching and coloration state. Generally, a high CE value is an indication of large optical modulation under small charge insertion. Figure 6(a–d) plots ΔOD at a wavelength of 633 nm as a function of the charge inserted into films. The CE value of the as-synthesized WO_3_/ITO/glass was estimated from the slopes of the quasi-linear curves. The CE values at 633 nm were as follows: 175 nm (79.8 cm^2^ C^−1^), 200 nm (90 cm^2^ C^−1^), 225 nm (77.5 cm^2^ C^−1^), and 250 nm (72.3 cm^2^ C^−1^).

### Characterization of WO_3_/NiO ECD

**Figure 7:**
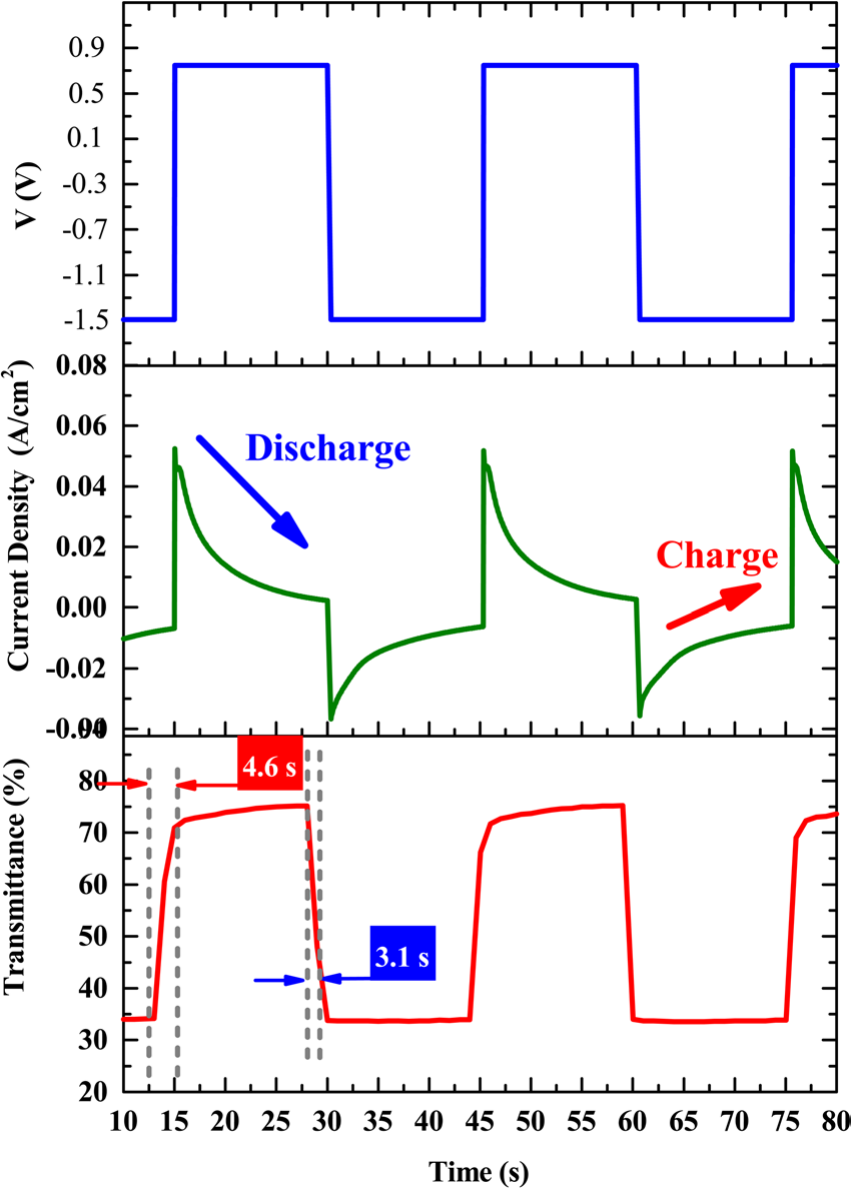
(Color online) (**a**) While the optical modulation for applied voltage of −1.5 V for colored state and 0.8 V for bleached state within interval of each step was controlled at 15 s; (**b**) corresponding CA curve of ECD; (**c**) switching time between bleached and colored states measured at 633 nm between 0.8 V and −1.5 V.

**Figure 8:**
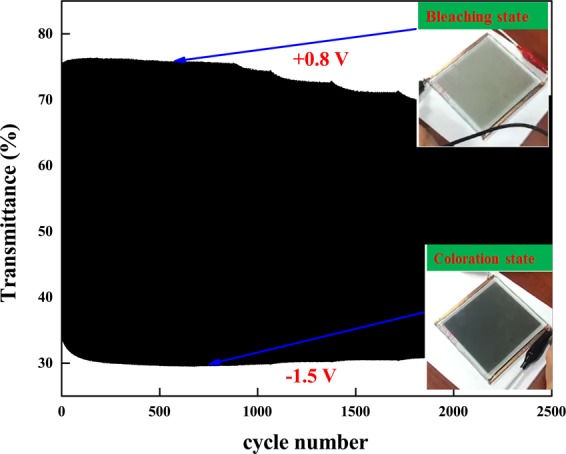
The durability of ECDs in terms of transmittance optical modulation at 633 nm following 2500-cycles.

Figures 7,8 presents the electrochromic performance of ECD (glass/ITO/WO_3_/liquid electrolyte/NiO/ITO/glass) with an active area of 3 × 4 cm^2^. Figure 7(a) presents the *in-situ* transmittance of WO_3_/NiO ECD at 633 nm, as analyzed during a continuous potential cycle from −1.5 *V* (colored potential, V_*c*_) to 0.8 *V* (bleached potential, V_*b*_). In Fig. 7(b,c) show that the coloration state (charge process) and bleaching state (discharge process) of ECDs were measured by CA curves and *in-situ* optical response of transmittance at fixed 633 nm. The coloration and bleaching of switching times or speed was a prominent characteristic for ECD system, which was defined as the time required for a 90% change in the full transmittance modulation^43–45^. As shown in Fig. 7(c), ECD achieved a maximum optical modulation reached 46% and the switching times at a wavelength of 633 nm were obtained coloration (3.1 sec) and bleaching (4.6 sec) (see supplementary video). The electrochromic and optical properties of our work compared with other authors researches are detailed in Table 3. Figure 8 illustrates the durability of the ECDs in terms of transmittance optical modulation measured in intervals of 15 s. As shown in Fig. 8, even after 1000 cycles (approximately 10 hours), there was no significant degradation in the optical modulation performance of the ECD. As shown in Fig. 8, transitions between bleached and colored states remained steady until 1000 cycles, at which point switching performance degraded gradually, dropping to 93% of the as-synthesized samples by 2500 cycles.

**Table 3:** exhibits the comparison of our results with the literature on various materials and methods^43–45,47–51^.

Materials/Device	Method	$\frac{\Delta {\rm{T}}}{( \% )}$	$\frac{{\rm{CE}}}{({{\rm{cm}}}^{2}/{\rm{C}})}$	$\frac{{\rm{Switchingtime}}}{({{\rm{t}}}_{{\rm{c}}}{/{\rm{t}}}_{{\rm{b}}})}$	Ref.
WO_3_/NiO	CAP	46	90	3.1/4.6 s	This work
Mo-doped WO_3_	RF sputtering	44.3	42.5	—	^24^
WO_3_/NiO	DC sputtering	55	87	10/20 s	^25^
WO_3_/PANI	Electropolymerization	37.4	98.4	9.9/13.6 s	^42^
WO_3_/MoO_3_	physicochemical	50	121.56	4.1 /3.4 s	^47^
WO_3_	Electrodeposition	88.51	137	3.7/5.2 s	^48^
WO_*x*_ nanorods	Exposure	57	33.3	11.8 /20.1 s	^49^
WO_3_ 0.33H_2_O/PEDOT	sol-gel	50.9	74.6	5/25 s	^50^
Tb-doped WO_3_	hydrothermal	66.71	48.33	3.7/9.99 s	^51^
WO_3_/PANI	dip-coating	54.3	79.7	1.4/1.1 s	^43^
WO_3_	spray	64	—	—	^44^
(NH_4_)_0.33_WO_3_	hydrothermal	51.6	60.9	5.7/4.2 s	^45^

## Methods

**Figure 9:**
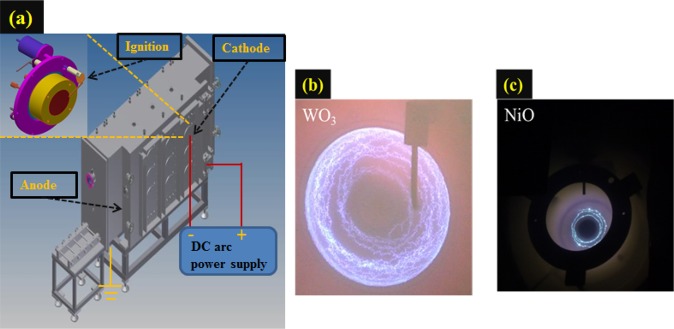
(**a**) Cathodic arc plasma (CAP) gun; (**b,c**) WO_3_/ NiO images showing the transient dynamics of cathodic spot motion.

Cathodic arcs can be used for the reactive deposition of various nitrides and oxides. Nonetheless, CAP technology has not been widely adopted, due to violent plasma-liquid pool interactions at cathode spots, which can cause the emission of macro-particles (MPs) that degrade the quality of the resulting film. This can largely be overcome by steering the arc rapidly across the surface of the cathode under high working pressure to reduce the spot residence time and limit the formation of erosion craters^46^. In recent years, researchers have shifted emphasis from monolithic coatings to higher performing multilayers and nano-composites. As shown in Fig. 9(a), the proposed arc gun set up relies on the flow of argon (for insertion) and oxygen (reaction) to control the formation of the electrode structure. It is difficult to measure the dynamics of a cathode spot; therefore, we employed a high-speed video camera to capture images of light emission from various spots across the target plane in sequences of 1 sec, as shown in Fig. 9(b,c). The deposition parameters are shown in Table 2 and 3, and the schematic drawing of the cathodic arc deposition is presented in Fig. 9(a).

**Figure 10:**
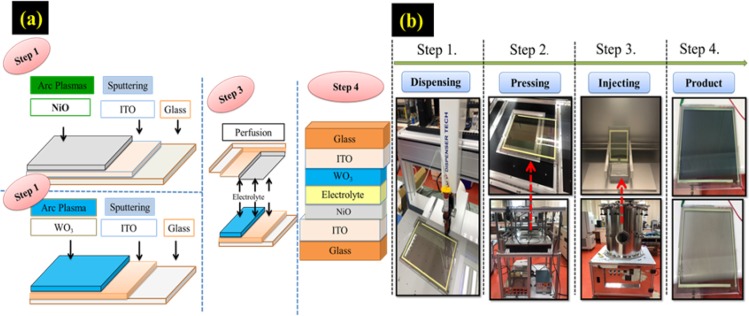
(**a**) Experimental procedure for Step1 to Step4. (**b**) Packaging process for ECD.

Figure 10 presents a schematic diagram showing the process of ECD fabrication. Figure 10(a): Step 1 and 2 involved respectively depositing an EC film/ITO on one glass substrate and a counter film/ITO on another glass substrate. Step 3 involved fitting the two components together and sealing them with epoxy adhesive. Note glass beads were used as spacers to maintain a cavity between the EC film and the counter film to hold liquid electrolyte (<100 *μ*m). Note also that a small gap was created in the epoxy for use as an inlet into the space. Step 5 showed ECD consisted of seven layers: A central ionic conduction layer (electrolyte) sealed between an electrochromic (EC) layer and an ion storage (complementary) layer, which were sandwiched between two transparent conducting layers, which were in turn sandwiched between two glass substrates. Figure 10(b) mainly describes the process of component packaging. Figure 10(b) Step 1: Dispensing for one side pre gluing. Step 2: UV glue curing. Step 3 involved filling the space between the two layers with liquid electrolyte ion injection in a vacuum pump. Step 4 production components.

### Synthesis of porous electrochromic and dense transparent electrodes films

Indium tin oxide (ITO, Solaronix SA, R_*sh*_ = 6.1 Ω/□)-coated glass was cut into wafers (5 × cm^2^) for use as a conducting substrate in ECDs. The ITO wafers underwent ultrasonic cleaning respectively in deionized water and ethyl alcohol for 2 min each. WO_3_/NiO films were deposited in series on the ITO glass using cathodic arc plasma (CAP) with targets of metallic tungsten (W) (99.95%) and Nickel (Ni) (99.95%) (76 mm in diameter and 12 mm in thickness) at room temperature, as shown Fig. 10(a). The base chamber pressure was maintained at less than 2 × 10^−5^ Torr using a turbo pump. Tables 4 and 5 list the fabrication parameters. CAP was used to deposit WO_3_/NiO as working/counter electrodes. As shown in the schematic in Fig. 10(a), the active area of the ECD (3 × 4 cm^2^) possessed the following structure: glass/ITO/WO_3_/liquid electrolyte/NiO/ITO/glass. Our primary focus in the current study was the analysis of WO_3_ films in terms of charge capacity and diffusion coefficient. The WO_3_/NiO series were fabricated on ITO glasses as electrochromic layers, which are listed in Tables 4 and 5.

**Table 4:** Details of WO_3_ processing parameters.

WO_3_ Pro.	$\frac{{\rm{W}}{\rm{.P}}.}{({\rm{Torr}})}$	$\frac{{\rm{Ar}}}{{{\rm{O}}}_{2}}$	$\frac{{\rm{DC}}\,{\rm{power}}}{({\rm{W}})}$	$\frac{{\rm{Time}}}{({\rm{\min }})}$	$\frac{{\rm{Dep}}.\,{\rm{Rate}}}{({\rm{nm}}\,/\,{\rm{\min }})}$	$\frac{{\rm{Dep}}.\,{\rm{Temp}}.}{^\circ \,C}$
Sample 1	8.3 × 10^−3^	0.2	1250	11	15.9	25
Sample 2	8.3 × 10^−3^	0.2	1250	13	15.4	25
Sample 3	8.3 × 10^−3^	0.2	1250	15	15	25
Sample 4	8.3 × 10^−3^	0.2	1250	17	14.7	25

**Table 5:** More details of ECD deposition parameters.

Target	$\frac{{\rm{W}}{\rm{.P}}.}{({\rm{Torr}})}$	$\frac{{{\rm{Ar}}/{\rm{O}}}_{2}}{({\rm{sccm}})}$	$\frac{{\rm{DC}}\,{\rm{power}}}{({\rm{W}})}$	$\frac{{\rm{Time}}}{({\rm{\min }})}$	$\frac{{\rm{Deposition}}.\,{\rm{Rate}}}{({\rm{nm}}\,/\,{\rm{\min }})}$	$\frac{{\rm{Dep}}.\,{\rm{Temp}}.}{^\circ \,C}$	$\frac{{\rm{Thick}}}{({\rm{nm}})}$
ITO	3 × 10^−3^	*Ar* = 100	500	60	5	200	300
Ni Metal	8.3 × 10^−3^	1/3(*Ar* = 120)	650	2.5	20	50	60

### Electrolyte layer

The liquid electrolyte system comprised lithium perchlorate (LiClO_4_, Mw = 106.39, Sigma-Aldrich, Darmstadt, Germany) and propylene carbonate (PC, C_4_H_6_O_3_, Sigma-Aldrich) at a weight ratio of 0.1325 (LiClO_4_/PC = 26.5 g/200 mL).

### Measurements

Electrochemical characterization was performed using cycle voltammetric (CV) and chronoamperometric (CA) (Autolab, model PGSTAT 30) measurements. *In–situ* UV-Vis measurements were obtained using a spectrophotometer (Ocean Optics, DH-4000-BAL) in conjunction with CA analysis.

## Conclusions

In conclusion, we have developed a CAP deposition as an alternative to sputtering in order to achieve high deposition rates with low-cost method of producing EC film based on WO_3_ for ECD applications. The proposed deposition scheme was applied to the synthesis of WO_3_ films with nanostructured surface features in various thicknesses (175 nm, 200 nm, 225 nm, and 250 nm). The complementary WO_3_(200 nm)/NiO (60 nm) ECD exhibits higher optical modulation (46% at 633 nm), faster response times (t_*b*_ = 4.6 *s*, t_*c*_ = 3.1 *s*) and higher CE (90 cm^2^/C). During the durability test, the transmittance change of ECD remained 43% after 2500 cycles, which was about 93% of original state.

## Supplementary information

Supplementary Video.
